# Parental Psychological Control and Adolescent Aggressive Behavior: Deviant Peer Affiliation as a Mediator and School Connectedness as a Moderator

**DOI:** 10.3389/fpsyg.2019.00358

**Published:** 2019-02-21

**Authors:** Yunlong Tian, Chengfu Yu, Shuang Lin, Junming Lu, Yi Liu, Wei Zhang

**Affiliations:** ^1^School of Education, Guangzhou University, Guangzhou, China; ^2^School of Education, Center for Brain and Cognitive Sciences, Guangzhou University, Guangzhou, China; ^3^Faculty of Social and Public Administration, Guangdong Baiyun University, Guangzhou, China; ^4^School of Psychology, South China Normal University, Guangzhou, China

**Keywords:** parental psychological control, deviant peer affiliation, school connectedness, aggressive behavior, adolescent development

## Abstract

Abundant evidence has demonstrated an association between parental psychological control and adolescent aggressive behavior. However, the mediating and moderating mechanisms underlying this relation are still under-investigated. Grounded in the social development model and stress-buffering model, this study investigated whether deviant peer affiliation mediates the relation between parental psychological control and adolescent aggressive behavior, and whether this indirect link is moderated by school connectedness. A total of 4265 adolescents (*Mean*_age_ = 13.66 years, *SD* = 2.74, 48.63% male) from southern China completed questionnaires regarding parental psychological control, deviant peer affiliation, school connectedness, and aggressive behavior. Structural equation models revealed that the relation between parental psychological control and aggressive behavior is partially mediated by deviant peer affiliation. Moreover, this indirect link was stronger for adolescents with low levels of school connectedness than for those with high levels of school connectedness. This study thus identifies the potential underlying mechanism by which parental psychological control is associated with adolescent aggressive behaviors, which has important implications for theory and prevention.

## Introduction

Aggressive behavior is a highly prevalent problem behavior that often occurs in adolescence. According to DSM-V, aggressive behavior is a widely prevalent symptom in psychiatric diseases, such as anxiety disorder, autism, and adjustment disorder (Takahashi et al., 2011). In the psychological studies, aggressive behavior has been generally defined as any behavior that has intention of causing harm to others who want to avoid being harmed (Anderson and Bushman, 2002; Bettencourt et al., 2006; Cabello et al., 2017). Furthermore, researchers describe aggressive behavior as defensive and premeditated (Takahashi et al., 2011). The previous studies found that aggressive behavior correlates significantly with several kinds of negative outcomes for both aggressors and victims of aggression, including tension and sleeping problems in aggressors, as well as depression and loneliness in victims (Becht et al., 2016; Cabello et al., 2017). Furthermore, early-life aggressive behavior is a risk factor for having serious problems with aggressive behavior in later life (Calkins and Keane, 2009). Therefore, understanding the development of aggressive behavior in adolescence is essential for the development of protective intervention programs.

Parental psychological control is an important negative parenting type that refers to parental control of the adolescent’s psychological world, and it includes tactics such as love withdrawal, devaluation, and guilt induction. The main aim of psychological control is to keep the adolescent emotionally dependent on the parents (Symeou and Georgiou, 2017). Ample research evidence has repeatedly shown that psychological control is a widely-used tactic in parenting, and it is an important predictor of adolescent aggressive behavior (Nelson et al., 2013; Cui et al., 2014). For instance, Nelson et al. (2013) found that parental psychological control predicts adolescent relational and physical aggression.

### Deviant Peer Affiliation as a Mediator

During adolescence, time spent with family steadily decreases and an increasing amount of time is spent in the company of peers (Zhu et al., 2016; Lin et al., 2018; Tian et al., 2019). Prior research has repeatedly shown that affiliate with deviant peers plays an important role in shaping adolescent problem behaviors including aggressive behavior (Longobardi et al., 2017; Zhu et al., 2017; Lin et al., 2018). According to the social development model (Hawkins and Weis, 1985; Choi et al., 2005), parental psychological control may promote the risk of adolescents affiliating with deviant peers, which in turn may increase adolescent delinquencies such as aggressive behavior. In line with the social development model (Hawkins and Weis, 1985), there is ample research evidence demonstrating the mediating role of deviant peer affiliation in the relation between negative parenting and adolescent problem behaviors including aggressive behavior (Hinnant et al., 2015; Zhu et al., 2017). For example, with a longitudinal study of aggression in Chinese students from grades 7 to 9, Zhu et al. (2017) reported that deviant peer affiliation mediated the effect of parental corporal punishment on adolescent physical aggression.

First, excessive psychological intervention by high-controlling parents may lead adolescents to acquire an interpersonal-control model of psychological control, which may force adolescents to affiliate with deviant peers (Cook and Fletcher, 2012; Zhu et al., 2017). This is due to parental psychological control having a negative effect on adolescent friendship, which has been observed within a wide range of adolescent friendliness, such as peer relation (Oudekerk et al., 2015) and peer rejection (Janssens et al., 2017). Meanwhile, poor social skills and negative relationship with conventional peers could positively predict deviant peer affiliation (Rudolph et al., 2014). Second, adolescents learn behavior patterns from their delinquent peers via observation and imitation, which in turn increase the risk of aggressive behavior (Bandura, 1977; Wang et al., 2017). Thus, parental psychological control may increase the risk of developing deviant peer affiliation through shape adolescents’ cognition of peer interaction (Cook and Fletcher, 2012; Zhu et al., 2017); which in turn would cause higher levels of aggression. Therefore, in this study, we propose the following hypothesis.

Hypothesis 1: Deviant peer affiliation will mediate the relationship between parental psychological control and adolescent aggressive behavior.

### School Connectedness as a Moderator

Although parental psychological control poses a crucial risk for adolescent aggressive behavior and deviant peer affiliation, adolescents can still achieve resilient positive outcomes. According to the stress-buffering model (Cohen and Wills, 1985), there are several protective factors that could mitigate the effect of environmental risk on the onset of aggressive behaviors. One such factor is school connectedness, which includes interactions between the adolescent and peers, between the adolescent and teachers, and between the adolescent and other significant members of the school (Fredricks et al., 2004). A few researchers have found that school connectedness moderates the impact of family factors. For example, Loukas et al. (2010) found a protective role of adolescent school connectedness against effects of negative parenting. In other words, school connectedness acts as a buffer for the negative effects of negative family relations on delinquent behaviors (Loukas et al., 2010). Other studies have found that school connectedness interacts with various social-environmental factors, such as parenting (Loukas et al., 2010) and peer factors (Millings et al., 2012; Rudasill et al., 2014; Oldfield et al., 2018), on adolescent behaviors. Further, other studies have shown that lower levels of school connectedness are positively correlated with aggressive behavior, while higher levels of school connectedness have important protective effects for adolescent development (Millings et al., 2012; Oldfield et al., 2018).

According to the stress-buffering model (Cohen and Wills, 1985), adolescents with strong school connectedness are more likely to accept the school’s norms, values, and expectations, and to refrain from aggressive behavior, given that problematic behaviors are inconsistent with the regulations of the school (Loukas and Pasch, 2013; Liu et al., 2016). Moreover, adolescents with high school connectedness may recognize the negative effects of psychological control and deviant peer affiliation, and they may be buffered by desire to obey the rules or fear of getting caught (Loukas and Pasch, 2013; Liu et al., 2016; Loke et al., 2016). Finally, school connectedness may be protective for adolescents under psychological control, by motivating them to conform to social regulations and by decreasing the likelihood of their engaging in deviant activities (Liu et al., 2016). Based on these theoretical suggestions and indirect experimental evidence, we expect that school connectedness would weaken the mediating effect of deviant peer affiliation toward the influence of psychological control on adolescent aggression. Therefore, we proposed the following hypothesis:

Hypothesis 2: School connectedness will moderate the indirect relation between parental psychological control and adolescent aggressive behavior. This indirect association will be stronger among adolescents with low levels of school connectedness but much weaker among adolescents with high levels of school connectedness.

### The Present Study

**FIGURE 1:**
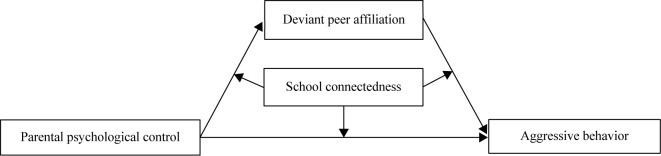
The conceptual model of the proposed moderated mediation framework.

As noted, previous research has examined the role of parental psychological control on aggressive behavior by adolescents, but less is known about the mediating and moderating mechanisms and the various factors affecting this relationship, such as deviant peer affiliation and school connectedness. Grounded in the social development model and stress-buffering model, this study investigated whether deviant peer affiliation mediates the relationship between parental psychological control and adolescent aggressive behavior, and whether this indirect link is moderated by school connectedness. Figure 1 illustrates the proposed research model.

## Materials and Methods

### Participant

The participants in this study were recruited from eleven schools in Guangdong province, southern China, through stratified and random cluster sampling. A total of 4265 adolescents (2074 boys) between the ages of 9 and 19 (*Mean_age_* = 13.66, *SD* = 2.74) participated. Reflecting the demographics of the sample, 54.35% of participants’ fathers and 62.49% of their mothers have less than a high school education; 39.22% come from rural areas, 43.20% from small-medium cities, and 17.58% from metropolitan areas. Moreover, 55.79% come from families with an average monthly income between ¥1000 to ¥5000, equivalent to about $148 to $738. The average personal monthly household income in China (2018) is ¥1753, equivalent to about $259.

### Measures

#### Parental Psychological Control

The eight-item Psychological Control Scale-Youth Self-report was used to measure the psychological control of participants’ parents (Barber, 1996). In this scale, adolescents report their perceptions of the extent to which parents have engaged in psychologically controlling behaviors (i.e., love withdrawal, devaluation, and guilt induction) in the past half year. A sample item is “My parents tell me that I should feel guilty when I do not meet their expectations.” Each item was rated on a scale from 1 (*Never*) to 3 (*Always*). The mean of the eight items was calculated, with higher scores reflecting higher parental psychological control. Cronbach’s *α* was 0.79.

#### Deviant Peer Affiliation

Adolescent reported on their deviant peer affiliation using the Chinese version of the Deviant Peers Questionnaire (Zhu et al., 2015). Twelve items that index deviant peer affiliation assessed how many of students’ peers had displayed deviant behaviors in the past half year. A sample item is “How many of your friends got involved in fights during the past six months?” Each item was rated on a scale from 1 (*Never*) to 3 (*Six or more times*). The mean of the 12 items was calculated, with higher scores reflecting higher deviant peer affiliation. Cronbach’s *α* was 0.88.

#### School Connectedness

The Emotional Engagement Subscale of the School Engagement Scale assessed adolescents’ school connectedness (Wang et al., 2011). This subscale consists of eight items, scored on a 5-point Likert scale (that is, from 1 = *Strongly disagree* to 5 = *Strongly agree*), and asks participants to report the extent of their connectedness with their school, with items such as “In general, I feel like a real part of this school.” The mean of the eight items was calculated, with higher scores reflecting higher school connectedness. Cronbach’s *α* was 0.76.

#### Aggressive Behavior

Participants reported on their own aggressive behaviors using the Chinese version of the Buss–Warren aggression questionnaire (BWAQ) (Maxwell, 2008; Lin et al., 2018). In the current study, 19 items were used indicating levels of physical, relationship, and verbal aggression behaviors during the past 6 months, such as “Once in a while, I can’t control the urge to strike another person.” Responses are rated on a 5-point scale (from 1 = *Not at all* to 5 = *Absolutely like me*). The mean of the 19 items was calculated, with higher scores reflecting more aggressive behavior. Cronbach’s *α* was 0.88.

### Procedure

Ethics approval for this study was obtained from the Certification of Ethics Review Committee of Education School, Guangzhou University. Written informed consent was obtained from the students’ teachers, all adult participants, and the parents/legal guardians of all non-adult participants. The data was collected by trained psychology teachers or graduate students in psychology. To encourage honest reporting, participants were assured that their answers would be kept confidential. They were requested to complete the anonymous questionnaires by themselves and were also told that they were free to withdraw any time during this study.

### Statistical Analyses

SPSS 20.0 was utilized for descriptive statistics. We used Mplus 7.1 to perform structural equation modeling in order to examine mediation and moderation effects (Muthén and Muthén, 1998–2012). In this study, less than 2% of data is missing, and the missing data was handled with full-information maximum likelihood estimation.

## Results

### Descriptive Statistics

Table 1 displays the means and standard deviations of the variables and the Pearson product-moment correlations for all variables in the current study. The results showed a significant, positive relation between parental psychological control and aggressive behavior. In addition, school connectedness was negatively associated with deviant peer affiliation and aggressive behavior, whereas deviant peer affiliation was positively associated with aggressive behavior.

**Table 1:** Descriptive statistics and the Pearson product-moment correlations for all variables.

Variable	*M*	*SD*	1	2	3	4
1. Parental psychological control	1.50	0.39	1.00			
2. School connectedness	3.78	0.64	-0.15^∗∗^	1.00		
3. Deviant peer affiliation	1.24	0.42	0.16^∗∗^	-0.15^∗∗^	1.00	
4. Aggressive behavior	1.73	0.53	0.25^∗∗^	-0.18^∗∗^	0.26^∗∗^	1.00


*^∗∗^p < 0.01.*

### Testing for a Mediation Effect

**FIGURE 2:**
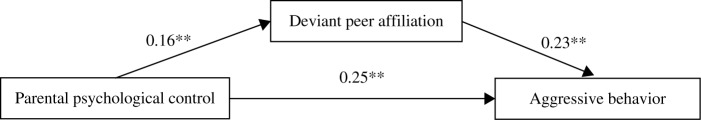
Model of the mediating role of deviant peer affiliation between parental psychological control and aggressive behavior. Nonsignificant paths, and paths between gender, age, SES (socioeconomic status was measured as the average of participant’s standardized scores reporting parental education level, parental occupation status, and family per capita monthly income; higher scores represent higher SES), and each of the variables in the model are not displayed. Of those paths, the following were significant: gender, age, and SES on deviant peer affiliation (*b*_1_ = 0.11^∗∗^, *b*_2_ = 0.01^∗∗^, *b*_3_ = -0.02^∗∗^, *b*_4_ = 0.05^∗∗^), and gender and age on aggressive behavior (*b*_1_ = 0.08^∗∗^, *b*_2_ = 0.02^∗∗^, *b*_3_ = 0.15^∗∗^). ^∗∗^*p* < 0.01.

The mediation model revealed that the model is identified to the data: *χ*^2^/*df* = 2.14, CFI = 0.99, RMSEA = 0.023. The results are displayed in Figure 2. Parental psychological control positively predicted deviant peer affiliation (*b* = 0.16, *SE* = 0.02, *t* = 9.78, *p* < 0.01) and positively predicted aggressive behavior (*b* = 0.25, *SE* = 0.02, *t* = 13.21, *p* < 0.01), while deviant peer affiliation also positively predicted aggressive behavior (*b* = 0.23, *SE* = 0.02, *t* = 12.40, *p* < 0.01). Moreover, bootstrapping analyses indicated that deviant peer affiliation partially mediated the relation between parental psychological control and aggressive behavior (indirect effect = 0.0352, *SE* = 0.0055, 95% CI = [0.0249, 0.0471]).

### Testing for Moderated Mediation

**FIGURE 3:**
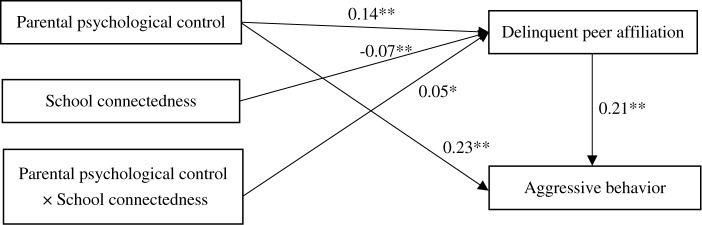
Model of the moderating role of school connectedness on the indirect relationship between parental psychological control and aggressive behavior. Nonsignificant paths, and between gender, age, SES, and each of the variables in the model are not displayed. Of those paths, the following were significant: gender, age and SES on delinquent peer affiliation (*b*_1_ = 0.10^∗∗^, *b*_2_ = 0.01^∗^, *b*_3_ = -0.02^∗∗^, *b*_4_ = 0.05^∗∗^), gender, age and SES on aggressive behavior (*b*_1_ = 0.08^∗∗^, *b*_2_ = 0.01^∗∗^, *b*_3_ = 0.15^∗∗^). ^∗^*p* < 0.05, ^∗∗^*p* < 0.01.

**FIGURE 4:**
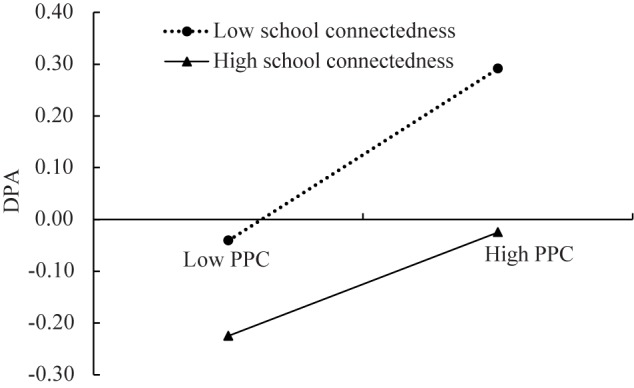
Deviant peer affiliation among adolescents as a function of parental psychological control and school connectedness. PPC, Parental psychological control; DPA, Deviant peer affiliation.

The moderated mediation model represented in Figure 3 had an excellent fit to the data: *χ^2^*/*df* = 2.69 CFI = 0.99, RMSEA = 0.034. The results showed that school connectedness moderated the association between parental psychological control and deviant peer affiliation (*b* = -0.05, *SE* = 0.02, *t* = -2.02, *p* < 0.05). We conducted a simple slopes test, and as depicted in Figure 4, the positive association between parental psychological control and deviant peer affiliation was much stronger for adolescents with lower school connectedness (*b* = 0.17, *SE* = 0.02, *t* = 7.82, *p* < 0.01) than for adolescents with higher school connectedness (*b* = 0.10, *SE* = 0.02, *t* = 4.65, *p* < 0.01). Moreover, parental psychological control was positively linked to aggressive behavior (*b* = 0.23, *SE* = 0.02, *t* = 12.14, *p* < 0.01), as was deviant peer affiliation (*b* = 0.21, *SE* = 0.02, *t* = 10.57, *p* < 0.01). However, there was no significant interaction between school connectedness and deviant peer affiliation in predicting aggressive behavior (*b* = -0.03, *SE* = 0.03, *t* = -1.15, *p* > 0.05).

The results revealed that conditional indirect effects were significant for adolescents with lower school connectedness (indirect effect = 0.0375, *SE* = 0.0079, 95% CI [0.0235, 0.0546]) and for those with higher school connectedness (indirect effect = 0.0196, *SE* = 0.0070, 95% CI = [0.0082, 0.0370]). Adolescents with lower school connectedness were more likely to associate with deviant peers, which in turn contributed to higher levels of aggressive behavior.

## Discussion

The purpose of this study was to investigate the mediating and moderating mechanisms underlying the relationship between parental psychological control and adolescent aggressive behavior. Based on the social development model and stress-buffering model, we hypothesized that deviant peer affiliation would mediate the relationship between parental psychological control and adolescent aggressive behavior, and that school connectedness would moderate the indirect association. By investigating these mechanisms, we would be able to identify effective family and school interventions for reducing the risk of adolescent aggressive behaviors.

First, this study found that adolescent aggression is affected by parental psychological control via deviant peer affiliation. This result is consistent with Hypothesis 1 and the social development model (Hawkins and Weis, 1985; Choi et al., 2005), indicating that experiencing psychological control from parents affects adolescents’ tendencies for aggressive behavior. When adolescents experience higher levels of psychological control from their parents, they are more likely to affiliate with deviant peers, which in turn promotes aggressive behavior. According to the social development model, adolescents with high levels of parental psychological control may acquire patterns of interpersonal-control (Cook and Fletcher, 2012; Zhu et al., 2017), which in turn may increase the likelihood of negative peer relationships such as deviant peer affiliation (Oudekerk et al., 2015). Furthermore, when adolescents affiliate with delinquent peers, they are more likely to exhibit aggressive behavior because of learning behavior patterns through processes of peer pressure, modeling, and norms (Bandura, 1977; Wang et al., 2017).

Second, this study found that the indirect link “parental psychological control → deviant peer affiliation → aggressive behavior” is stronger for adolescents with low school connectedness than adolescents with high school connectedness. This finding indicates that school connectedness interacts with a parenting factor (parental psychological control) to amplify the mediating processes. Further, the results indicate that school connectedness only moderates the first stage of the mediating effect (i.e., parental psychological control → deviant peer affiliation). This finding is partially consistent with our hypothesis 2 and the stress-buffering model (Cohen and Wills, 1985), in which strong school connectedness protects against deviant peer affiliation under psychological control, presumably because strong school connectedness motivates adolescents to conform to social regulations and decreases the likelihood of their involvement in deviant peer groups (Loukas et al., 2010; Liu et al., 2016). These findings show that school connectedness decreases the influence of the parental psychological control on deviant peer affiliation in adolescents (Loukas et al., 2010). However, the moderating effects of school connectedness on relations between parental psychological control, deviant peer affiliation, and adolescent aggressive behavior are not significant. This could be because such connectedness principally reflects their relationships with others at school (Millings et al., 2012). Therefore, it could buffer the adverse effects concerning social process (i.e., deviant peer affiliation) but could not buffer behavioral outcomes of parental psychological control. In addition, a number of empirical studies have shown that deviant peer affiliation robustly predicts adolescent problem behaviors (Li et al., 2016; Ding et al., 2017), therefore, school connectedness may not be enough to buffer these adverse effects. Future studies are needed to further find important moderators between deviant peer affiliation and adolescent development.

## Implications and Limitations

This study examined the mediating and moderating mechanism between parental psychological control and adolescent aggressive behavior in a large sample. These findings provide some targeted intervention suggestions for reducing the risk of adolescent aggressive behavior. First, we provide evidence that parental psychological control might increase the risk of adolescent aggressive behavior. These results suggest that parents should avoid controlling their adolescents’ psychological world in family life, as it might cause them to develop aggressive behavior. Second, consistent with the social development model (Hawkins and Weis, 1985; Choi et al., 2005), this study demonstrated the important mediating role of deviant peer affiliation. This result suggests that parents and teachers should provide a positive model of friendship (Cook and Fletcher, 2012; Zhu et al., 2017), and they should help adolescents to develop positive relationship with their friends (Bandura, 1977; Wang et al., 2017), in order to decrease their risk for adopting aggressive behavior. Third, we investigated why some adolescents, despite exposure to parental psychological control, do not show high levels of aggressive behavior. Our results suggest that adolescents with high levels of connectedness with their school may buffer their risk for adopting aggressive behavior. These findings suggest that school educators can effectively help adolescents (especially those with high parental psychological control) avoid developing aggressive behavior by increasing their connectedness with the school.

Several limitations should be considered in the interpretation and generalizability of the present findings. First, even though we used a large sample to test the moderation and mediation models of adolescent problem behavior, our cross-sectional design questionnaire method does not permit us to establish a causal direction. Future studies should use longitudinal designs or other methods to verify the causal relationships. Second, all data was reported by adolescents, who may not have been fully informed in assessing information about parental psychological control and peer affiliation. We need to be cautious about possible bias, including self-presentation biases (Williams et al., 1989; Longobardi et al., 2018; Settanni et al., 2018). Future studies should also include data reports from parents, peers, and teachers, in order to elicit more accurate information. Third, in this study, we have focused on several factors to explain the mediating and moderating mechanisms of adolescent aggressive behavior. However, there are also other factors that have important roles in adolescent aggressive behaviors, such as parental corporal punishment, social status of adolescents, and student-teacher relationships (Zhu et al., 2017; Longobardi et al., 2018). Finally, previous research has shown that the development of adolescent aggressive behavior is significantly affected by disorders such as ADHD (Farbiash et al., 2014), autism (Singh et al., 2011), depression (Marsh et al., 2016), and so on. Therefore, further research needs to include these factors as independent variables or control variables to better understand the etiology of adolescent aggressive behavior.

## Author Contributions

YT, CY, and WZ conceived and designed the research. YT and CY performed the research. YT, CY, and SL analyzed the data. YT, CY, and SL contributed to the writing of the manuscript. YT, CY, SL, JL, YL, and WZ revised the paper critically for important intellectual content, commented on, and approved the final manuscript.

## Conflict of Interest Statement

The authors declare that the research was conducted in the absence of any commercial or financial relationships that could be construed as a potential conflict of interest.
